# Acceptance Rate of COVID-19 Vaccine and Its Determinants Among Indian Pregnant Women: A Hospital-Based Cross-Sectional Analysis

**DOI:** 10.7759/cureus.30682

**Published:** 2022-10-25

**Authors:** Archana Kumari, Suman Kumari, Manisha Kujur, Sarita Tirkey, Shashi Bala Singh

**Affiliations:** 1 Obstetrics and Gynecology, Rajendra Institute of Medical Sciences, Ranchi, IND; 2 Obstetrics and Gynecology, Rajendra Institute of Medical sciences, Ranchi, IND; 3 Preventive and Social Medicine, Rajendra institute of Medical Sciences, Ranchi, IND; 4 Obstetrics and Gynecology, Rajendra institute of Medical Sciences, Ranchi, IND

**Keywords:** india, determinants, acceptance, pregnant women, vaccine, covid-19

## Abstract

Background

Vaccination is more widespread when the determinants and hesitancy of vaccination are identified, especially in vulnerable groups, such as pregnant women. Pregnant women if infected with COVID-19 are more likely to get severe COVID-19 illness and adverse neonatal outcomes as compared to non-pregnant women. The present study was designed with the aim to estimate the rate of COVID-19 vaccine acceptance and to identify the associated factors which influence the vaccine acceptance.

Methods

This study was a hospital-based cross-sectional study conducted in the Obstetrics Department of Rajendra Institute of Medical Sciences, Ranchi, Jharkhand, from February 2022 to April 2022. Our study included 298 pregnant women above 18 years who were willing to participate in the study. Information was collected by face-to-face interview using a structured and pretested questionnaire. Binomial logistic regression (univariate and multivariate) was used to identify the associated factors on vaccination acceptance.

Results

Among 298 pregnant women, 234 expressed willingness to receive vaccine, giving an acceptance rate of 78.52%. The educational status, ethnicity, occupation of the woman and husband, and type of family showed no significant relationship with the vaccine acceptance. On multivariate logistic regression analysis, the determinants found to be significantly associated with COVID-19 vaccine acceptance among pregnant women were as follows: Muslim religion (aOR=0.27, CI: 0.12-0.61), gravida >2 (aOR=1.84, CI: 1.30-2.61), and awareness that COVID-19 vaccine has been approved by the government (aOR=3.03, CI: 1.45-6.36). Awareness that COVID-19 infection causes more severe complications in pregnant women than non-pregnant women (aOR=1.89, CI: 0.93-3.87) and hypertension (aOR=0.36, CI: 0.11-1.20) were non-significantly associated.

Conclusion

The acceptance of COVID-19 vaccination was high in this study and was well received especially by mothers who had knowledge about the importance of vaccination during pregnancy. Concerns about the side effects of vaccination and the possibility of harming the baby were the main reasons for refusal. During prenatal care, health care providers should reinforce the benefits of COVID-19 vaccination during pregnancy.

## Introduction

The impact of coronavirus disease 2019 (COVID-19) in terms of global mortality and morbidity has been huge [1]. Globally, there have been 539,893,858 confirmed cases of COVID-19, including 6,324,112 deaths, as reported to World Health Organization (WHO) till June 23, 2022 [2]. Effective preventive measures include COVID-19 appropriate behavior such as social distancing, use of face masks, and personal hygiene; however, the most crucial strategy for containing the COVID- 19 pandemic over the long term is vaccination-induced herd immunity [3]. Vaccination is more widespread if the determinants and hesitancy of vaccination are identified, especially in vulnerable groups, such as pregnant women.

Pregnancy does not increase the risk of COVID-19 infection, but if infected, pregnant women are more likely to get severe COVID-19 illness requiring hospitalization, intensive care unit (ICU) admission, and mechanical ventilation, and to die from illness compared to non-pregnant women [4,5]. Pregnant women with comorbid conditions such as obesity, diabetes, and hypertension are more likely to get severe COVID illness. The adverse neonatal outcomes especially preterm birth and low birth weight are also more likely in pregnant women with COVID-19 illness compared to pregnant women without COVID-19 infection [6].

International professional groups uniformly support the COVID vaccination in pregnancy and breastfeeding since the danger of contracting COVID-19 in pregnancy and the associated morbidity is significantly greater than any potential risks from the vaccine [7,8]. The Society for Maternal-Fetal Medicine and the American College of Obstetricians and Gynecologists (ACOG) have recommended that vaccine should be offered to pregnant and lactating women [9,10].

Pregnant and lactating women were excluded from COVID-19 vaccination in the initial rollout of two vaccines available in India - Covishield (nonreplicating viral vector vaccine) and Covaxin (inactivated virus). Furthermore, on recommendation of the National Technical Advisory Group on Immunization (NTAGI), Ministry of Health and Family Welfare (MoHFW), Government of India approved COVID-19 vaccination of pregnant women in July 2021. This decision empowers pregnant women to make an informed choice to take COVID-19 vaccine.

A study reported low acceptance of COVID-19 vaccination (37%) in a sample of pregnant women, and concern about vaccine safety was the major reason for hesitancy [11]. In a questionnaire-based survey from 16 countries, 52.0% of pregnant women expressed willingness to accept COVID-19 vaccine [12].

To our knowledge, there is no study conducted to assess the rate of COVID-19 vaccine acceptance among Indian pregnant women in the state of Jharkhand. Therefore, the present study was designed with the aim to estimate the rate of COVID-19 vaccine acceptance and to identify the associated factors that influence the vaccine acceptance. An understanding of the challenges and determinants of vaccine acceptance would further aid the acceleration of vaccine administration within these populations.

## Materials and methods

This was a hospital-based cross-sectional study conducted in the antenatal clinic of the Department of Obstetrics and Gynecology at Rajendra Institute of Medical Sciences, Ranchi, Jharkhand, from February 2022 to April 2022 after approval from the Institutional Ethics Committee (IEC number 03, dated 24.01.2022).

Sample size was calculated using the following formula: n = Z2xP (1-P)/m2 (Z=95% confidence level, P=prevalence, m=margin of error). Considering the percentage of pregnant women attending the Outpatient Department (OPD) of Obstetrics and Gynecology to be 24% as per previous records, 95% confidence interval (CI), and a margin of error at 5%, the sample size came out be 280. However, during the study period, we were able to collect data from 298 pregnant women.

Study population comprised all pregnant women attending the antenatal clinic during the data collection period. Pregnant women above 18 years of age who were willing to participate in this study were included after taking informed consent. Pregnant women with a history of mental illness, hearing loss, seriously ill or with some emergency conditions, and being unwilling to participate were excluded from the study. Women who had received vaccine in the past were also excluded from the study.

 Method of sampling was consecutive, and every pregnant women who came to the antenatal clinic to receive antenatal services and met the inclusion criteria was interviewed (20-30 cases per day twice a week). After obtaining informed consent, information was collected by face-to-face interview (which lasted for about 4-5 minutes) using a structured and pretested questionnaire. The questionnaire was developed and validated by opinion from experts and a pilot study. Each expectant woman underwent a private interview with strict confidentiality after receiving routine antenatal services. Data collectors and pregnant women were informed to follow the COVID-19 prevention protocols such as using face mask, maintaining physical distancing, and using hand sanitizer during data collection time. The filled questionnaires were collected and checked for completeness and consistency by investigators. The questionnaire was divided in three sections. The first section of the questionnaire included demographic information and general health status such as age, gravidity, parity, educational status, ethnicity, residence, religion, occupation, and family status. The second section evaluated the perception of risk related to COVID-19 pandemic. The third section focused on acceptance attitude of COVID-19 vaccine and reasons of non-acceptance.

Vaccine acceptance was defined as a response of not receiving vaccine in the past but willingness to receive COVID-19 vaccine during pregnancy, whereas vaccine non-acceptance was defined as a response of not taking vaccine in past and not willing to receive vaccine during pregnancy.

Statistical analysis

The data were collected, coded, entered, and analyzed using Stata MP 13. Categorical data are described as number and percentage, Chi-square test was used to test and describe the relationship between two categorized variables. Binomial logistic regression (univariate and multivariate) was used to test the predictors of the binary outcome variable. Variables that were significantly associated with COVID-19 vaccine acceptance in a univariate analysis were further entered into a multivariate logistic regression analysis. The adjusted odds ratio (OR) and 95% CI were used to know the odds of COVID-19 vaccine acceptance for each significant variable. A p-value of 0.05 was considered significant.

## Results

**Figure 1:**
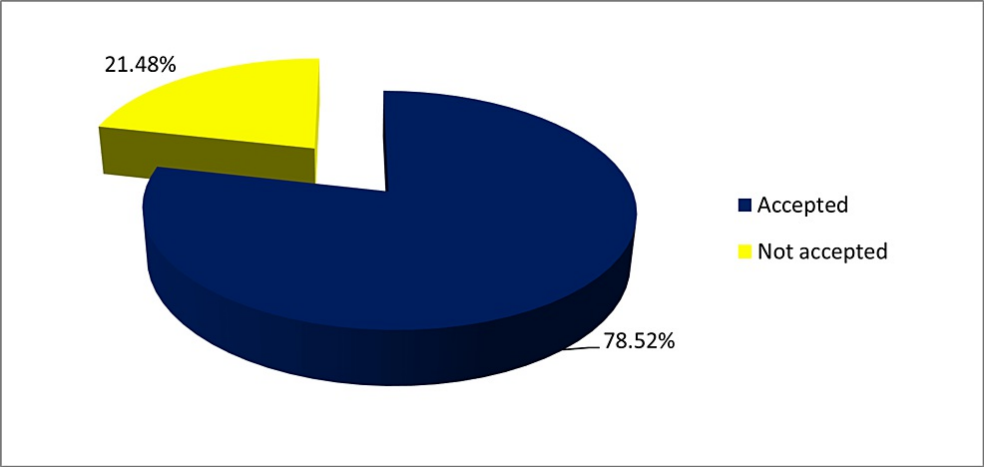
COVID vaccine acceptance rate among pregnant women (n=298)

In this study, a total of 298 pregnant women participated. Among these, 234 (78.52%) expressed willingness to receive COVID-19 vaccine, while 64 (21.48%) refused to take COVID-19 vaccine, as shown in Figure 1.

Table 1 shows the sociodemographic characteristics of pregnant women and willingness to accept COVID-19 vaccine. Among the participants willing to receive vaccine, majority (73.93%) belonged to the age group of 21-30 years, 80.77% were from urban areas, 83.33% were of non-tribal ethnic groups, 76.5% followed Hindu religion, and 77.78% were unemployed. Regarding education status, only 6.41% were illiterate, and the remaining had at least primary level of education. Awareness about COVID-19 complications in pregnant women was a significant factor, as 68.8% of pregnant women who had this knowledge were willing to receive the vaccine.

**Table 1:** Sociodemographic characteristics of pregnant women and acceptance of COVID-19 vaccination Data are presented as number (%) H/o, history of; HTN, hypertension

Variable	Acceptance of COVID-19 vaccine		p- Value
	Not willing to receive COVID-19 vaccine (n=64), number (%)	Willing to receive COVID-19 vaccine (n=234), number (%)	
Age (years)			0.500
18-20	6 (9.38)	30 (12.82)	
21-30	51 (79.69)	173 (73.93)	
31-40	6 (9.38)	31 (13.25)	
>40	1 (1.56)	0	
Residence			0.676
Rural	15 (23.44)	45 (19.23)	
Urban	49 (76.56)	189 (80.77)	
Ethnicity			0.971
Tribal	10 (15.63)	39 (16.67)	
Non-Tribal	54 (84.38)	195 (83.33)	
Religion			0.208
Hindu	42 (65.63)	179 (76.50)	
Muslim	14 (21.88)	25 (10.68)	
Sikh	0	1 (0.43)	
Christian	5 (7.81)	18 (7.69)	
Sarna	3 (4.69)	11 (4.70)	
Educational status			0.889
Illiterate	3 (4.69)	15 (6.41)	
Primary	14 (21.88)	56 (23.93)	
Matriculation	17 (26.56)	54 (23.08)	
Intermediate	13 (20.31)	54 (23.08)	
Graduate and above	17 (26.56)	55 (23.50)	
Occupation of pregnant women			0.297
Unemployed	47 (73.44)	182 (77.78)	
Daily wage worker	9 (14.06)	15 (6.41)	
Private job	8 (12.50)	20 (8.55)	
Government job	0	13 (5.56)	
Self-employed	0	4 (1.71)	
Occupation of husband			0.087
Unemployed	1 (1.56)	13 (5.56)	
Daily wage worker	12 (18.75)	64 (27.35)	
Private job	28 (43.75)	89 (38.03)	
Government job	13 (20.31)	45 (19.23)	
Self-employed	10 (15.63)	23 (9.83)	
Type of family			0.116
Nuclear	20 (31.25)	101 (43.16)	
Joint	44 (68.75)	133 (56.84)	
Gravida			0.195
≤2	61 (95.31)	205 (87.60)	
>2	3 (4.69)	29 (12.39)	
Number of live children			0.270
0	8 (12.50)	48 (20.51)	
≤2	50 (78.13)	165 (70.51)	
>2	6 (9.38)	21 (8.97)	
Comorbid conditions			0.405
Diabetes	3 (4.60)	7 (2.99)	
Heart disease	2 (3.10)	7 (2.99)	
HTN	7 (10.93)	13 (5.55)	
Respiratory disease	1 (1.56)	6 (2.56)	
Others	8 (12.50)	17 (7.26)	
None	43 (67.18)	184 (78.63)	
H/o COVID-19 infection in past in you?			0.648
No	50 (78.13)	172 (73.39)	
Yes	14 (21.88)	62 (26.61)	
Did you follow preventive measures for COVID-19 infection?			0.865
No	9 (14.06)	31 (13.25)	
Yes	55 (85.94)	203 (86.75)	
H/o COVID-19 infection in family members?			0.090
No	49 (76.56)	153 (65.38)	
Yes	15 (23.44)	81 (34.62)	
H/o adverse effects of COVID-19 vaccine in family members?			0.842
No	54 (84.38)	195 (83.33)	
Yes	10 (15.63)	39 (16.67)	
Awareness regarding COVID-19 vaccine approval in India?			0.254
No	40 (62.50)	93 (39.74)	
Yes	24 (37.50)	141 (60.26)	
Awareness that COVID-19 infection causes more severe complications in pregnant women than in non-pregnant?			0.004
No	34 (53.12)	73 (31.20)	
Yes	30 (46.87)	161 (68.80)	

The associations between acceptance toward the COVID-19 vaccine and sociodemographic data, history of COVID-19 infection in past, preventive measures taken, side effects of COVID-19 vaccine in family members, and knowledge about importance of COVID-19 vaccine are shown in Table 2 (univariate regression analysis). We found that among all the variables, the following ones were significantly associated with the acceptance of COVID-19: Muslim religion (p=0.02, OR: 0.41; CI: 0.20-0.87), awareness that COVID-19 vaccine has been approved by the government (p=0.001, OR: 2.52; CI: 1.42-4.46), and awareness that COVID-19 infection causes more severe complications in pregnant women than non-pregnant women (p=0.003, OR: 2.42; CI: 1.36-4.30). Significant association was not observed with other variables.

**Table 2:** Univariate logistic regression analysis for determinants of COVID-19 vaccine acceptance OR, odds ratio; CI, confidence interval; HTN, hypertension

Variables		p-Value	OR	95% CI for OR	
				Lower	Upper
Residence	Rural	Ref			
	Urban	0.17	1.28	0.69	7.8
Ethnicity	Tribal	Ref			
	Non-tribal	0.84	0.92	1.01	17.45
Religion	Hindu	Ref			
	Muslim	0.02	0.41	0.20	0.87
	Christian	0.75	0.84	0.29	2.40
	Sarna	0.82	0.86	0.22	3.22
Educational status	Illiterate	Ref			
	Primary	0.75	0.80	0.20	3.15
	Matriculation and above	0.57	0.69	0.19	2.49
Occupation of patient	Unemployed	Ref			
	Employed	0.46	0.78	0.41	1.48
Occupation of husband	Unemployed	Ref			
	Employed	0.21	0.26	0.03	2.10
Gravida	≤2	Ref			
	>2	0.10	2.77	0.81	9.44
Number of live children	0	Ref			
	≤2	0.29	0.54	0.17	1.69
	>2	0.48	0.55	0.10	2.85
Family	Nuclear	Ref			
	Joint	0.08	1.67	0.92	3.00
Comorbid conditions	None	Ref			
	Diabetes	0.50	0.62	0.15	2.49
	Heart disease	0.95	0.95	0.19	4.71
	HTN	0.13	0.47	0.18	1.25
	Respiratory disease	0.64	1.65	0.19	14.02
	Others	0.18	0.54	0.22	1.33
COVID-19 infection in past (Yes)		0.44	1.29	0.66	2.50
Followed preventive measures (Yes)		0.86	1.07	0.48	2.38
Family members had COVID-19 infection (Yes)		0.09	1.72	0.91	3.27
Family had adverse effects after COVID-19 vaccine (Yes)		0.84	1.06	0.50	2.30
Aware that COVID-19 vaccine has been approved by the government for pregnant women (yes)		0.001	2.52	1.42	4.46
Aware that pregnant women have more risk to develop complications of COVID-19 infection than non-pregnant women (yes)		0.003	2.42	1.36	4.30

In multivariate logistic regression analysis (Table 3), the most significant associations between COVID-19 vaccine acceptance and the variables were Muslim religion (aOR=0.27, CI-0.12-0.61), gravida > 2 (aOR=1.84, CI-1.30-2.61), and awareness that COVID-19 vaccine has been approved by the government (aOR=3.03, CI: 1.45-6.36). Hypertension (aOR=0.36, CI-0.11-1.20) and awareness that COVID-19 infection causes more severe complications in pregnant women than non-pregnant women (aOR=1.89, CI: 0.93-3.87) were non-significantly associated.

**Table 3:** Multivariate analysis of factors associated with COVID-19 vaccination during pregnancy aOR, adjusted odds ratio; CI, confidence interval

Variables	aOR	95% CI	p-Value
Religion: Muslim	0.27	0.12-0.61	0.002
Gravida >2	1.84	1.30-2.61	0.001
History of hypertension	0.36	0.11-1.20	0.09
Aware that COVID-19 vaccine has been approved by the government for pregnant women	3.03	1.45-6.36	0.003
Aware that pregnant women have more risk to develop complications of COVID-19 infection than non-pregnant women	1.89	0.93-3.87	0.07

**Figure 2:**
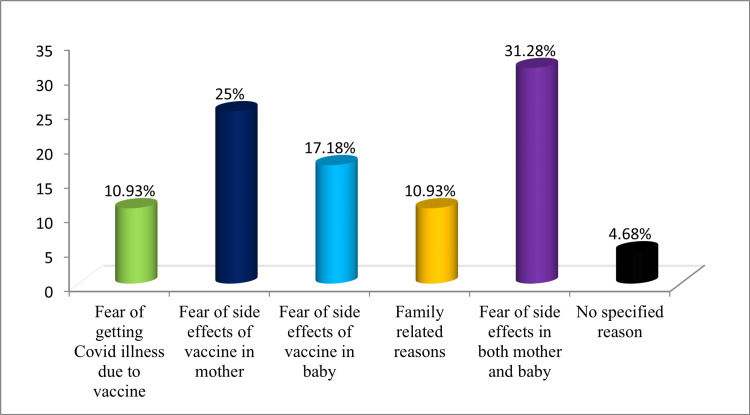
Reasons for refusal of COVID-19 vaccine acceptance (n=64)

The reasons for refusing COVID-19 vaccine have been depicted in Figure 2. The most common reasons were fear of harm due to side effects of vaccine in both the mother and the baby (31.2%), followed by fear of harm due to side effects of vaccine in the mother (25%). Fear of getting disease due to vaccine and family hesitancy was also cited as the reasons in 10.9% each.

**Figure 3:**
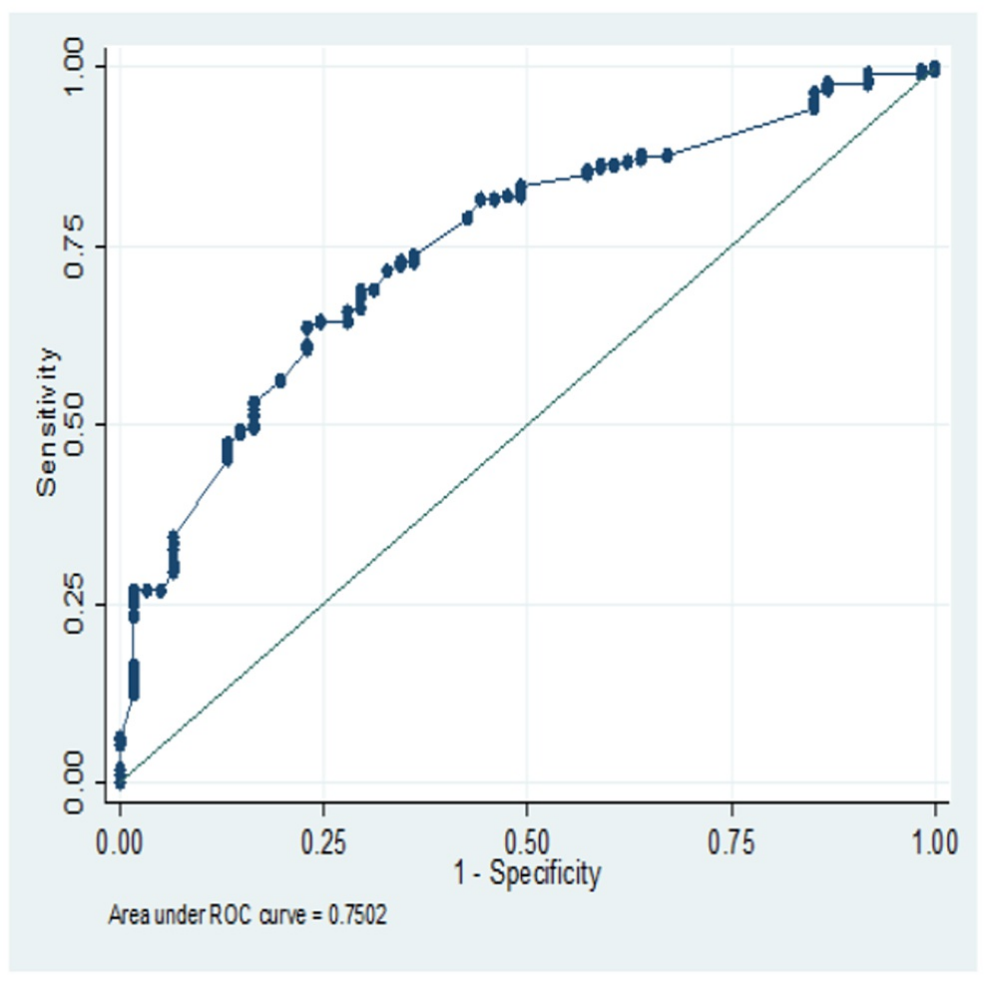
Receiver operating characteristic (ROC) analysis

The logistic multivariable model has 75.02% predictive accuracy of the vaccine acceptance (Figure 3).

## Discussion

During the pandemic period, the demand for COVID-19 vaccine has been strong and acceptance was high among Jharkhand population, while the main hindrance in promotion of vaccine uptake was concerns of vaccine safety. Vaccination can be said to be effective only when it is highly accepted and used. The acceptance rate of COVID-19 vaccine among pregnant women in our study was high with 78.52% willing to receive COVID-19 vaccine. This is in contrast to previous studies that reported low acceptance rate of 37%, 52%, 41%, 44.3%, and 58.3% [11-15]. Rate of willingness to be vaccinated for COVID-19 during pregnancy was 60% among Thai women in study by Pairat and Phaloprakarn and 68% in a study by Ghamri et al. from Saudi Arabia [16,17]. However, the acceptance rate in our study is similar to a study from China by Tao et al., which showed a high acceptance rate 77.4% [18]. These results are also consistent with acceptance rate of vaccine in the general population (74%) in a study from France [19]. The differences in vaccine acceptance rate among pregnant women could be attributed to study population differences, varying awareness of COVID-19 severity, risk perception, and access to health care services.

In univariate logistic regression, we found no association of educational status, ethnicity, occupation of the woman and husband, type of family, with the vaccine acceptance. This finding is similar to a Thai study, which also found no association of socioeconomic status, education, and occupation, with COVID-19 vaccine acceptance [16]. On the contrary, higher income and older age were strong predictors of vaccine acceptance in a survey from 16 countries [12]. Our finding is also different from a study by Ghamri et al., which showed that educated women are more likely to receive COVID-19 vaccine than uneducated women [17]. However, in our study, women following Muslim religion were less willing to receive COVID-19 vaccine than women following other religions. The reasons for this seem unclear, but may be related to less awareness about vaccine availability and concern about vaccine safety.

Women who were aware that the COVID-19 vaccine has been approved by the government, as well as the fact that pregnant women have higher risk of developing severe complications following COVID-19 infection than non-pregnant women, were more willing to receive the vaccine than those who were unaware of these facts. These pregnant women with good knowledge were approximately 2.5 times more likely to accept COVID-19 vaccination. This can possibly be explained as pregnant women with good knowledge about COVID-19 disease may be aware of the severity of COVID-19 infection to both themselves and their baby for them to readily accept the COVID-19 vaccine to reduce the impact of the pandemic. Maternal knowledge about COVID-19 vaccine is thus a significant driver of vaccine uptake.

Multivariate analysis indicated that awareness regarding approval of vaccine among pregnant women by the government is associated with a threefold increase in vaccine acceptance. This result reflects the faith of women among government policies and trust on scientific approval of the vaccine for the benefit of pregnant women. Multivariate analysis also indicated that women who are pregnant for third time or more are twice more likely to receive COVID-19 vaccination. This is probably due to higher concern about care of their children in the event of getting COVID-19 infection.

In our study, 21.48% pregnant women refused to take vaccine. The most common reason for refusal to take vaccine was fear of side effects in both the mother and the baby, followed by fear of side effects in mothers themselves. Fear of getting COVID-19 disease due to vaccine and family hesitancy was also cited as the reasons. These findings are similar to a study conducted by Goncu Ayhan et al. and Ghamri et al. [11,17]. This can be explained as a natural fear of the mother about her pregnancy and concerns about safety of the vaccine. Other than natural fear, there may be various other contributory factors, such as the psychological status of mothers, source of information about COVID-19 vaccination from peer discussion, television, and social media, and other information. Thus, there is a need to spread awareness about potential advantages and safety evidence of vaccination during pregnancy, which would help the mothers to make an informed decision about acceptance of vaccine. Recommendations from family members, friends, and doctors can also aid in pregnant women getting vaccinated.

The major strength of this study is the moderate sample size with multiple study variables. But our study is limited by its cross-sectional design, as such results cannot show temporal association between variable and effect.

## Conclusions

The acceptance of COVID-19 vaccination in pregnant women was high and was well received especially in mothers who had knowledge about the importance of vaccination during pregnancy. Concerns about the side effects of vaccination and the possibility of harming the baby were the main reasons for refusal. During prenatal care or outpatient consultation, health care providers should explain the benefits of COVID-19 vaccination during pregnancy. There is a need to increase the public awareness about safety of COVID-19 vaccine during pregnancy.
